# Looking Beyond the Lens of Crimean-Congo Hemorrhagic Fever in Africa

**DOI:** 10.3201/eid3007.230810

**Published:** 2024-07

**Authors:** Olalekan John Okesanya, Gbolahan Deji Olatunji, Emmanuel Kokori, Noah Olabode Olaleke, Olaniyi Abideen Adigun, Emery Manirambona, Don Eliseo Lucero-Prisno

**Affiliations:** Neuropsychiatric Hospital Aro, Abeokuta, Nigeria (O.J. Okesanya, O.A. Adigun);; University of Ilorin, Ilorin, Nigeria (G.D. Olatunji, E. Kokori);; Kwara State University, Malete, Nigeria (N.O. Olaleke);; Obafemi Awolowo University Teaching Hospital Complex, Ile-Ife, Nigeria (N.O. Olaleke);; University of Rwanda, Kigali, Rwanda (E. Manirambona);; London School of Hygiene and Tropical Medicine, London, UK (D.E. Lucero-Prisno III)

**Keywords:** Crimean-Congo hemorrhagic fever, CCHF, CCHF virus, CCHFV, outbreak, ticks, vector-borne infections, viruses, zoonoses, Africa

## Abstract

Crimean-Congo hemorrhagic fever (CCHF) is a lethal viral disease that has severe public health effects throughout Africa and a case fatality rate of 10%–40%. CCHF virus was first discovered in Crimea in 1944 and has since caused a substantial disease burden in Africa. The shortage of diagnostic tools, ineffective tick control efforts, slow adoption of preventive measures, and cultural hurdles to public education are among the problems associated with continued CCHF virus transmission. Progress in preventing virus spread is also hampered by the dearth of effective serodiagnostic testing for animals and absence of precise surveillance protocols. Intergovernmental coordination, creation of regional reference laboratories, multiinstitutional public education partnerships, investments in healthcare infrastructure, vaccine development, and a One Health approach are strategic methods for solving prevention challenges. Coordinated efforts and financial commitments are needed to combat Crimean-Congo hemorrhagic fever and improve all-around readiness for newly developing infectious illnesses in Africa.

Crimean-Congo hemorrhagic fever (CCHF) is caused by a tickborne virus belonging to the genus *Nairovirus* within the family Bunyaviridae. The disease was first observed among military personnel from the former Soviet Union who were stationed in Crimea during World War II (*1*), leading to the name Crimea hemorrhagic fever. Later, researchers discovered that the virus found in Crimea was the same as the Congo virus, which caused febrile illness in the Belgian Congo. Therefore, the virus was named Crimean-Congo hemorrhagic fever virus (CCHFV). The virus was identified after it was isolated from the blood and tissues of infected persons and intracerebral inoculation of suckling mice (*2*). Since its identification, CCHFV has been recognized as a major public health threat. Outbreaks have been reported across numerous countries, particularly in Africa, the Middle East, Asia, and parts of Europe (*1*,*2*).

CCHFV is transmitted to humans through tick bites, direct contact with the blood from an infected person, or contact with blood or tissue from infected livestock. Tickborne transmission typically occurs through the bite of hard-bodied *Hyalomma* ticks belonging to the family Ixodidae (*2*). However, >35 tick species, including several soft ticks from the family Argasidae, are carriers of CCHFV (*1*,*3*). Some of those ticks are carried across Europe and other areas by migratory birds following flight paths across continents. Among the tickborne viruses that affect humans, CCHFV has the broadest geographic distribution.

Persons at greatest risk for exposure to infected ticks are those who engage in outdoor activities, such as forest workers, farmers, soldiers, and hikers. Persons at greatest risk for exposure to the blood or tissue of infected livestock are veterinarians, farmers, shepherds, slaughterhouse employees, and butchers (*4*,*5*). CCHFV can also be transmitted through human-to-human sexual contact, intrafamily transmission, or aerosol-producing medical procedures (*6*,*7*) and other nosocomial routes. Household contacts of infected patients and healthcare personnel are at risk for exposure through contact with patient blood and secretions or needlesticks (*8*). CCHFV transmission is increased by climatic and environmental changes, increased tick populations, livestock movement, and transport of virus-infected ticks by migratory birds (*9*).

The incubation period for CCHF can range from 9 days after a tick bite to 14 days after direct contact with the blood of an infected person or livestock (*3*). The disease progresses through 4 stages: incubation, prehemorrhagic, hemorrhagic, and convalescent stages. The prehemorrhagic stage is characterized by high fever, muscle pain, severe headaches, chills, nausea, vomiting, diarrhea, facial redness, and rash. As the disease advances, subcutaneous bleeding and hemorrhagic symptoms occur in various organs of the body. CCHF-positive patients often have low lymphocyte, monocyte, and neutrophil counts (*10*,*11*). The disease has a high case-fatality rate of 10%–40% (*1*).

Since the initial report of CCHF in Crimea in 1944, sporadic outbreaks of the disease have been reported intermittently in Europe, Asia, and Africa (*12*); the highest number of outbreaks have been reported in the former Soviet Union, Turkey, Iran, Bulgaria, China, and Africa (*13*,*14*). Although CCHF is mostly undiagnosed and unreported in Africa, the continent has a high CCHF disease burden; 62 cases and 17 deaths occurred in outbreaks during 2003–2018 (*10*). Africa has experienced several outbreaks with varying rates of cases and deaths in Uganda, Senegal, South Africa, and Mauritania (*10*). In a 2023 outbreak in Namibia, the index patient was reported to have exhibited symptoms on May 16 and died on May 18; CCHF was confirmed by an unspecified laboratory method (*15*). We describe the status of CCHF in Africa, challenges for its control, and recommendations to mitigate CCHFV spread.

## CCHF Outbreaks in Africa

CCHF in Africa has likely been underestimated for decades because of shortcomings in surveillance (*16*). Through organized efforts between individual countries’ health departments and international organizations, such as the Africa Centre for Disease Control and the World Health Organization, 19 nations in Africa have recorded CCHF cases in humans from January 1956, when the first cases were reported, through July 2020 (*17*,*18*). During that period, >494 cases and 115 fatalities were recorded (*16*). Incidence for most of those nations was calculated according to random sampling or serosurveys, methods that are not sufficient to confirm the initial identification of CCHF cases (*19*). CCHF cases are underestimated in Africa because of a lack of active zoonotic surveillance and a dearth of appropriate diagnostic tools (*19*,*20*).

**Figure 1:**
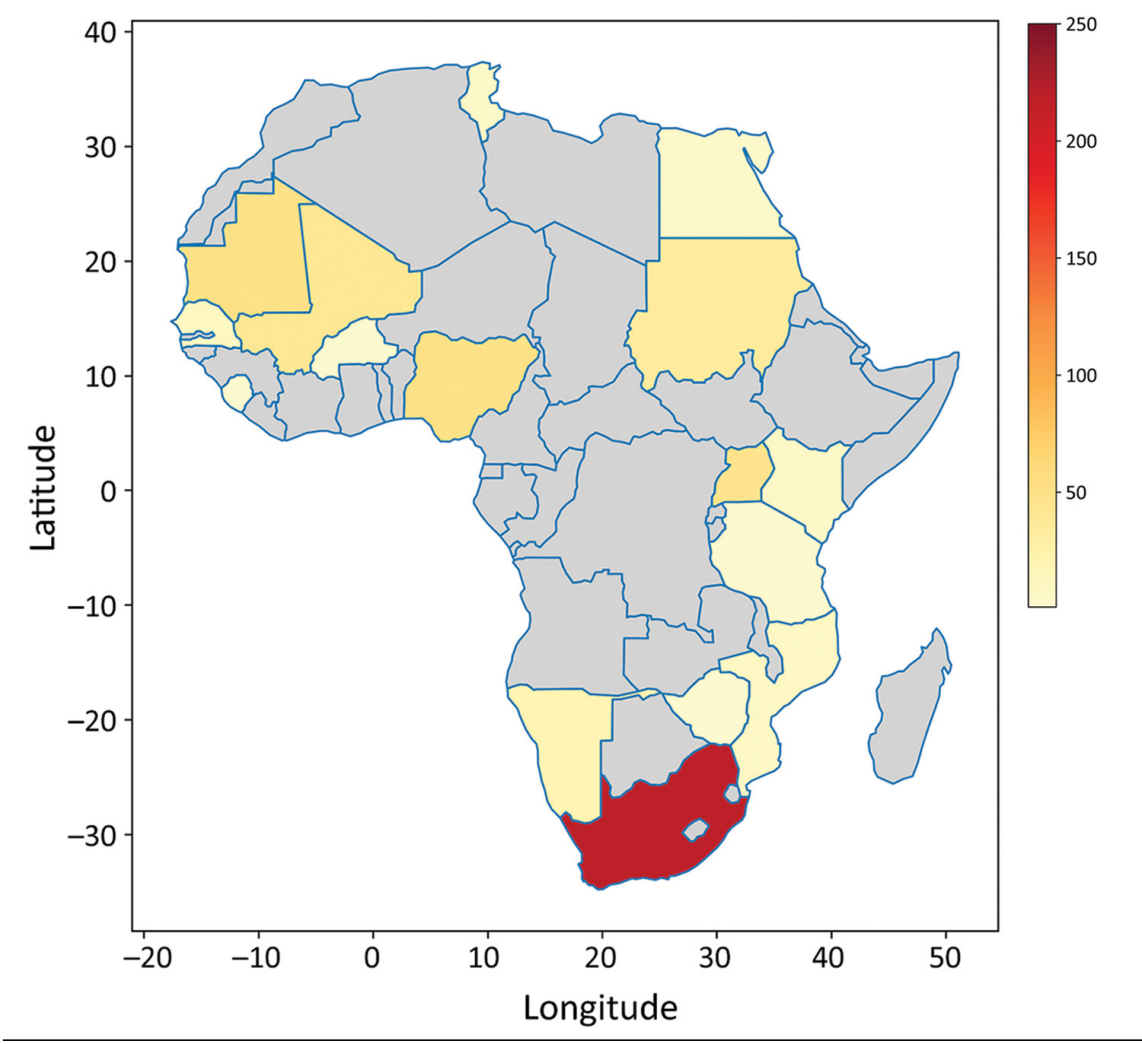
Distribution of Crimean-Congo hemorrhagic fever cases in Africa. Total numbers of CCHF cases were recorded by 19 nations in Africa during January 1956–July 2020. Colors in key indicate the number of confirmed cases in each country. Red indicates the highest number of cases occurred in South Africa. Gray indicates no cases were reported in those countries.

**Figure 2:**
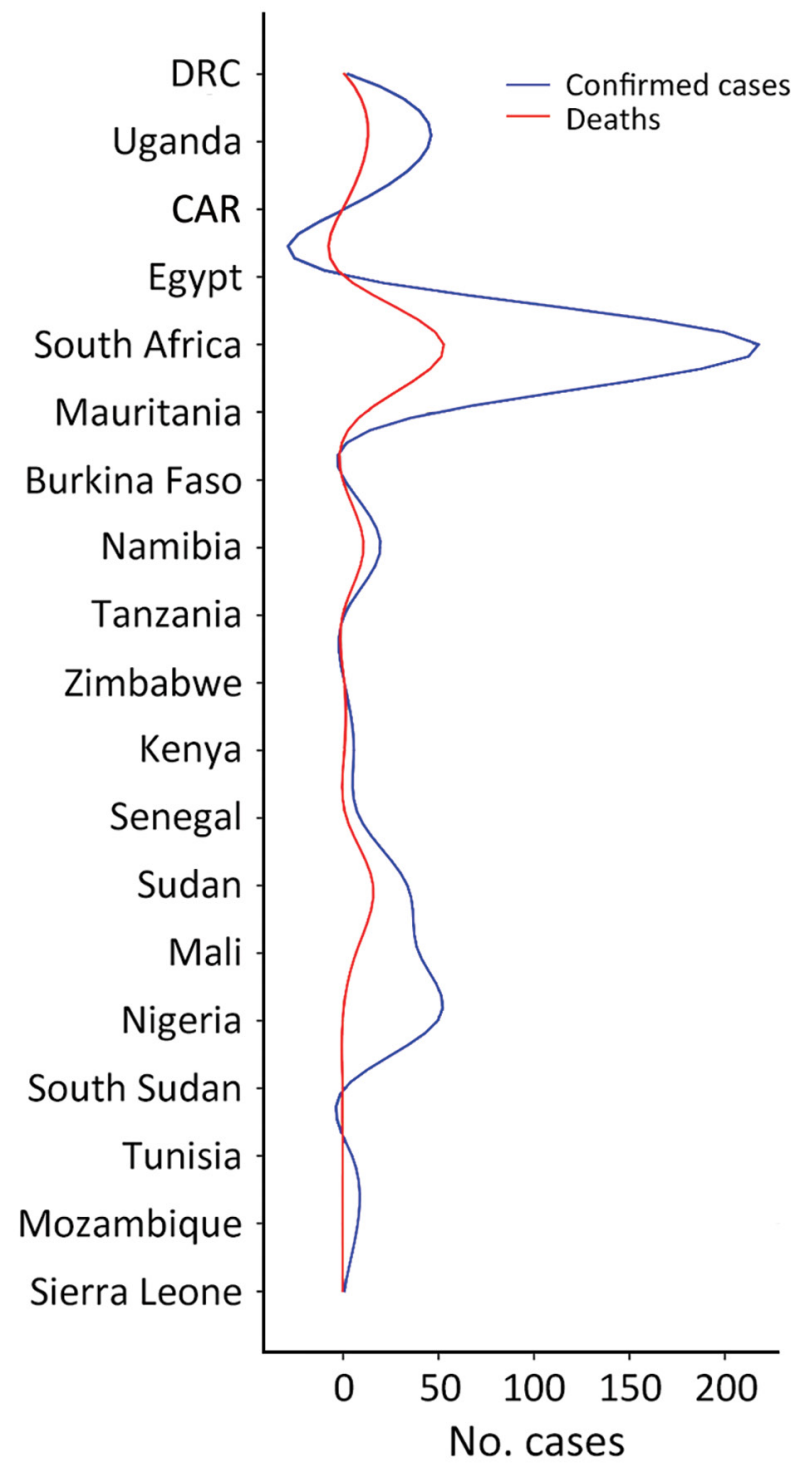
Number of Crimean-Congo hemorrhagic fever cases and related deaths in different countries within Africa. Total numbers of CCHF cases and deaths were recorded by 19 nations in Africa during January 1956–July 2020. CAR, Central African Republic; DRC, Democratic Republic of the Congo.

South Africa and Uganda have reported an appreciable number of CCHF cases and deaths because they established robust surveillance systems, the Special Pathogen Unit of the National Institute of Communicable Diseases in South Africa and the Virus Research Institute in Uganda (*16*,*21*). However, Mozambique, Eswatini, Botswana, and Lesotho have recorded no cases (*22*,*23*), and Tanzania, South Sudan, Zimbabwe, and Kenya have only confirmed 1 case each. No other cases have been recorded in Burkina Faso since its first reported case in 1983 (*24*). If other nations had developed robust surveillance systems, such as those in South Africa and Uganda, more cases would likely have been detected (Figures 1, 2) (*25*).

## Special Challenges Faced by Countries in Africa

Countries in Africa face special challenges in preventing and controlling CCHF. For example, those countries often lack efficient surveillance systems that are essential for early CCHF case detection and rapid outbreak response. Time delays from initial healthcare contact to diagnosis can lead to an increase in transmission (*26*). Patients have had to travel long distances to receive proper care (*15*,*27*). Insufficient collaboration between animal and human disease control sectors in Africa has posed a major obstacle to reducing outbreaks. In addition, the shortage of diagnostic laboratory kits for CCHF (particularly at the district level), the lack of accessible diagnostic facilities, inadequate laboratory capacities, and a shortage of trained laboratory personnel have hindered the diagnostic process and response efforts. Those problems have led to misdiagnosis, delayed treatment, and an increase in fatalities (*16*). Another problem has been poor tick control activities, especially during animal-gathering events. The absence of tick control initiatives has likely exacerbated outbreaks, particularly in rural areas where resources are scarce.

Countries in Africa face obstacles in adopting personal protective measures among persons at risk for CCHF, such as healthcare workers and livestock handlers. The limited availability of protective equipment impedes compliance with preventive measures (*28*). Another challenge is certain cultural beliefs that thwart disease prevention and promote mistrust and low health literacy, which have reduced community participation and cooperation (*26*). Other obstacles include the lack of suitable serodiagnostic tests for extensive animal testing, the absence of clear guidance for standardized surveillance in the animal health sector, and the costs associated with implementing routine testing and surveillance (*29*,*30*).

## Recommendations for Africa

### Intergovernmental Assistance and Cross-Organizational Alliances

To strengthen the response to CCHF in Africa, collaboration between governments and institutions at the regional and continental levels is crucial. Such collaborations should enable development of human and ecologic surveillance networks for CCHF across the continent (*16*). During CCHF outbreaks, countries that have diagnostic capabilities should offer rapid diagnostic support to countries that do not have them. The have and have-not countries should work collectively to establish surveillance networks to control outbreaks and create a more comprehensive understanding of CCHF epidemiology in Africa. Collaborative efforts should include active surveillance, testing ticks for CCHFV, and conducting serologic tests for animals and humans with suspected CCHFV infections (*31*–*33*).

### Establishing Regional Reference Laboratories

The general lack of healthcare infrastructure in many countries in Africa poses challenges in investigating CCHF outbreaks, identifying cases promptly, isolating patients, and providing medical care. We recommend establishing regional reference laboratories in major geopolitical zones that are easily accessible by rural communities (*11*,*12*). Those laboratories should be equipped with standard diagnostic equipment that enable molecular assays, such as real-time PCR, which is the most accurate method of CCHF diagnosis; immunological assays, such as ELISA to identify specific IgG and IgM; and simple rapid tests. We also recommend enhancing other resources, such as laboratory diagnostic training plans for all health personnel, which could provide rapid diagnostic assistance during CCHF epidemics (*2*). Developing and using external quality assessment programs should be given top priority, and they should be used at the national or regional levels with the goals of increasing competence of medical specialists, standardizing diagnostic procedures, and guaranteeing the accuracy of CCHF test results (*34*,*35*). To enable effective diagnosis of CCHF by healthcare providers in Africa, we recommend external quality assessments and diagnostic evaluation methods, collaboration with international organizations, continual monitoring of diagnostic capabilities, and acquiring sufficient funds for those endeavors wherever possible (*36*). Better diagnostic capability would help improve the capacity for timely detection and response to CCHF cases in Africa, which is a crucial component of patient management and CCHFV transmission prevention (*37*).

### Multiinstitutional Collaboration for Public Education

Engaging communities in outbreak response activities, including education, risk communication, and behavioral change, is central to control CCHF outbreaks. Institutional collaboration in countries in Africa should prioritize public education on CCHFV transmission, including how the virus is transmitted and the importance of using personal protective equipment (PPE), when recommended. It is essential to provide appropriate PPE to persons at risk for CCHF, such as animal handlers and healthcare workers, and to educate them on how to use the PPE effectively while practicing good hygiene to prevent both community and nosocomial outbreaks (*38*). Collaborative efforts can contribute to reducing the spread of CCHFV and safeguarding public health by raising awareness and ensuring access to necessary protective gear (*39*).

### Investment in Public Health Infrastructure

Countries in Africa need to increase investments in public health infrastructure, such as by strengthening staff capacity, upgrading healthcare facilities, and establishing well-equipped laboratories. Improving training programs and raising awareness are needed to develop a skilled public health workforce that can respond effectively to epidemics (*40*). We recommend additional support for research projects across the continent to better understand the epidemiology and transmission dynamics of CCHF and to predict, prevent, and manage outbreaks (*39*). Further research is also needed to explore potential vaccines and evaluate the efficacy of treatment options, including ribavirin and other antiviral drugs.

### Vaccines

CCHF is the most common tickborne viral disease infecting humans, calling for effective vaccines to prevent outbreaks and reduce transmission risks (*41*). Several vaccine options are being researched and developed with promising results. However, no widely accepted CCHF vaccines are available for humans or animals. Preclinical trials have shown promise for various vaccine candidates, including attenuated live virus vaccines, vectored vaccines (using virus vectors to deliver CCHFV antigens), and plant-expressed vaccines (producing CCHFV antigens in plants for vaccine delivery). Those options are being investigated to determine their efficacy and potential as preventive strategies against CCHF. To optimize CCHF vaccine efficacy and investigate other therapeutic approaches, comprehensive detailed research, including preclinical research, clinical trials, and continuous surveillance, is required (*42*). Since 1974, Bulgaria has used the Bulgarian vaccine, an inactivated vaccine made from the brain tissue of suckling mice infected with CCHFV (*43*). The vaccine is given subcutaneously, and persons >16 years of age need several booster doses. T-cell activity against CCHFV is strong after vaccination, but persons receiving numerous doses also exhibit higher levels of IgG and interferon-secreting effector T cells (*43*,*44*). Neutralizing antibody levels in immunized persons are still insufficient even after booster shots. Despite being used domestically, the Bulgarian vaccine has not been approved globally because of safety concerns and the lack of controlled clinical efficacy trials (*41*), emphasizing the continuous need for further research on and development of CCHF vaccines. To address the genetic diversity of various CCHFV strains in vaccine development, it is essential to optimize vaccine formulations, evaluate dosing regimens, and identify immunogenic epitopes that confer robust and lasting immunity against CCHFV (*45*). Identifying those epitopes is critical for developing vaccines that can defend against CCHFV genetic variants (*15*). As previously described, inactivated vaccines produced from mouse brains have been used, but they are not widely available. Developing new mouse models that simulate human CCHF disease has proven to be beneficial in vaccine research (*46*). In addition to human immunization initiatives, veterinary vaccines for livestock have the potential to minimize exposure during animal handling and disrupt the tick-vector cycle. Despite continuous research, no animal vaccines are available on the market; however, the modified vaccinia virus Ankara glycoprotein GP vaccine is being tested in sheep, which represents an essential area of investigation (*3*,*29*,*47*).

### One Health Approach

The World Health Organization introduced a One Health approach to improve coordination among livestock and agriculture professionals, veterinarians, and researchers. The approach brings together agriculture, environmental service, and veterinary service sectors to achieve better CCHF control and prevention and further development of preventive practices, research, and training in Africa (*48*). The One Health approach acknowledges the need for a comprehensive and interdisciplinary strategy to control CCHF epidemics in Africa (*49*).

## Conclusions

CCHF imposes a substantial disease burden in Africa. Public education, tick control, infection prevention and control, and protection for persons conducting high-risk activities, such as livestock rearing and working in slaughterhouses, are essential in CCHF-endemic areas to protect populations at risk for CCHFV infection. In addition, developing reliable diagnostics, effective vaccines, and antiviral treatments is crucial for minimizing the burden of CCHF on patients and healthcare systems. Sustained commitment and cooperation among nations of Africa, regional organizations, and international partners that prioritize collaborative efforts, strengthen surveillance networks, and invest in public health infrastructure will safeguard public health and produce a continent better prepared to effectively respond to CCHF and other disease outbreaks.
